# Structural Characterization of a Neutralizing Nanobody With Broad Activity Against SARS-CoV-2 Variants

**DOI:** 10.3389/fmicb.2022.875840

**Published:** 2022-06-02

**Authors:** Tingting Li, Bingjie Zhou, Zhipu Luo, Yanling Lai, Suqiong Huang, Yuanze Zhou, Yaning Li, Anupriya Gautam, Salome Bourgeau, Shurui Wang, Juan Bao, Jingquan Tan, Dimitri Lavillette, Dianfan Li

**Affiliations:** ^1^State Key Laboratory of Molecular Biology, Chinese Academy of Sciences Center for Excellence in Molecular Cell Science, Shanghai Institute of Biochemistry and Cell Biology, Chinese Academy of Sciences, Shanghai, China; ^2^University of CAS, Beijing, China; ^3^CAS Key Laboratory of Molecular Virology and Immunology, Institut Pasteur of Shanghai CAS, Shanghai, China; ^4^Institute of Molecular Enzymology, School of Biology and Basic Medical Sciences, Soochow University, Suzhou, China; ^5^College of Pharmacy, Chongqing Medical University, Chongqing, China; ^6^Nanjing Crycision Biotech Co., Ltd., Nanjing, China; ^7^Institut National de la Santé et de la Recherche Médicale, École des Hautes Etudes en Santé Publique, Institut de Recherche en Santé, Environnement et Travail, Université de Rennes, Rennes, France; ^8^Pasteurien College, Soochow University, Suzhou, China

**Keywords:** conformation competition, coronavirus, COVID-19, crystal structure, nanobody, receptor-binding domain, SARS-CoV-2

## Abstract

SARS-CoV-2 and its variants, such as the Omicron continue to threaten public health. The virus recognizes the host cell by attaching its Spike (S) receptor-binding domain (RBD) to the host receptor, ACE2. Therefore, RBD is a primary target for neutralizing antibodies and vaccines. Here, we report the isolation and biological and structural characterization of a single-chain antibody (nanobody) from RBD-immunized alpaca. The nanobody, named DL28, binds to RBD tightly with a *K*_D_ of 1.56 nM and neutralizes the original SARS-CoV-2 strain with an IC_50_ of 0.41 μg mL^−1^. Neutralization assays with a panel of variants of concern (VOCs) reveal its wide-spectrum activity with IC_50_ values ranging from 0.35 to 1.66 μg mL^−1^ for the Alpha/Beta/Gamma/Delta and an IC_50_ of 0.66 μg mL^−1^ for the currently prevalent Omicron. Competition binding assays show that DL28 blocks ACE2-binding. However, structural characterizations and mutagenesis suggest that unlike most antibodies, the blockage by DL28 does not involve direct competition or steric hindrance. Rather, DL28 may use a “conformation competition” mechanism where it excludes ACE2 by keeping an RBD loop in a conformation incompatible with ACE2-binding.

## Introduction

A key step for SARS-CoV-2 infection is the molecular engagement between the receptor-binding domain (RBD) on the Spike (S) protein and the human receptor angiotensin-converting enzyme 2 (ACE2; Hoffmann et al., 2020; Shang et al., 2020; Walls et al., 2020; Wrapp et al., 2020a). The S is a heavily glycosylated trimeric protein that in the pre-form contains 1,273 amino acid residues. Upon cleavage by host proteases, S breaks down to two subunits, S1 and S2 at a region near residue 685. RBD (residues 330–526) is contained in the S1 subunit (Hoffmann et al., 2020). In the pre-fusion state, S exists in multiple conformations regarding the relative position of RBD to the rest of the protein. In its “closed” conformation, all three subunits are very similar and the receptor-binding motif (RBM) of the RBD is buried by adjacent N-terminal domains (NTDs) of S1. The RBD in the closed S is referred to as the “down” conformation and they are incompetent to engage with ACE2. In the “open” state, one, two, or all three RBDs could assume the “up” conformation, exposing the RBM to engage with ACE2 (Henderson et al., 2020; Walls et al., 2020; Wrapp et al., 2020a; Zhang et al., 2021). Reflecting the importance of ACE2-RBD binding in viral infection, hundreds of existing neutralizing antibodies target this event by direct blockage, steric hindrance, or locking the RBDs in the “down” conformation (Barnes et al., 2020).

The single-chain camelids-derived antibodies possess attractive features (Muyldermans, 2013). The variable region of the heavy-chain antibodies is referred to as nanobodies owing to their small sizes (~14 kDa). Despite having a single chain, nanobodies can target antigens with comparable selectivity and affinity to conventional antibodies. Being small, nanobodies are ultra-stable, relatively easy to produce (in microbial) with low costs and high yields, and amenable to protein engineering, such as fusion in various forms. Such fusion can result in improved potency, functional affinity and neutralizing activity can increase by hundreds to thousands of folds (Schoof et al., 2020; Li et al., 2021a; Yao et al., 2021). In addition, nanobodies that recognize non-competing epitopes can be conveniently fused to make biparatopic nanobodies that are potentially more tolerant to escape mutant strains (Koenig et al., 2021; Li et al., 2021a; Yao et al., 2021). The heat stability of nanobodies opens the possibility of using them as inhaling drugs for respiratory diseases (Muyldermans, 2013) [and indeed potentially for SARS-CoV-2 as demonstrated in hamsters Nambulli et al., 2021] and offers convenience in storage and transport. In the past months, dozens of neutralizing nanobodies against SARS-CoV-2 have been reported (Chi et al., 2020; Custódio et al., 2020; Esparza et al., 2020; Hanke et al., 2020; Huo et al., 2020a; Schoof et al., 2020; Xiang et al., 2020; Koenig et al., 2021; Li et al., 2021a; Pymm et al., 2021; Walter et al., 2022).

A challenge in developing neutralizing antibodies and vaccines against viruses is their ability to mutate. In particular, mutations in RBD that retain its structural integrity and function (ACE2-binding) may escape neutralizing antibodies by altering the binding surface either in composition or in conformation, or both (Weisblum et al., 2020; Harvey et al., 2021; Liu et al., 2021; Starr et al., 2021). In the past months, strains, such as the lineage B.1.1.7, B.1.351, P.1, B.1.617.2, B.1.1.529, referred to as the Alpha, Beta, Gamma, Delta, and Omicron variant by the World Health Organization, have caused outbreaks and concerns about how these variants, the Omicron in particular (Viana et al., 2022), could change the course of the pandemic due to their high virulence and their general resistance against antibodies and vaccines that were developed using previous strains (Hoffmann et al., 2021; Planas et al., 2021; Wang et al., 2021; Bolze et al., 2022). Given the large number of active cases, it is reasonable to assume that more escape mutants are almost certain to emerge. Due to the lag phase between outbreaks caused by new mutants and the development of vaccines/mAbs against the mutants, it is important to have different antibodies and to test and develop strategies to identify antibodies with broad reactivity and different neutralization mechanisms.

Here, we report the selection and structural characterization of an RBD-targeting neutralizing nanobody (dubbed DL28) isolated from immunized alpaca. The DL28 shows a broad activity against five variants of concern (VOCs) including the currently prevalent Omicron. Unlike most neutralizing nanobodies, the DL28 does not use direct competition or steric hindrance to block ACE2. Rather, structural analysis suggests a mechanism hypothesis by which DL28-binding causes a loop within the RBM to assume a conformation that is incompatible with ACE2-binding.

## Results

### Isolation of a High-Affinity Neutralizing Nanobody From Immunized Alpaca

**Figure 1:**
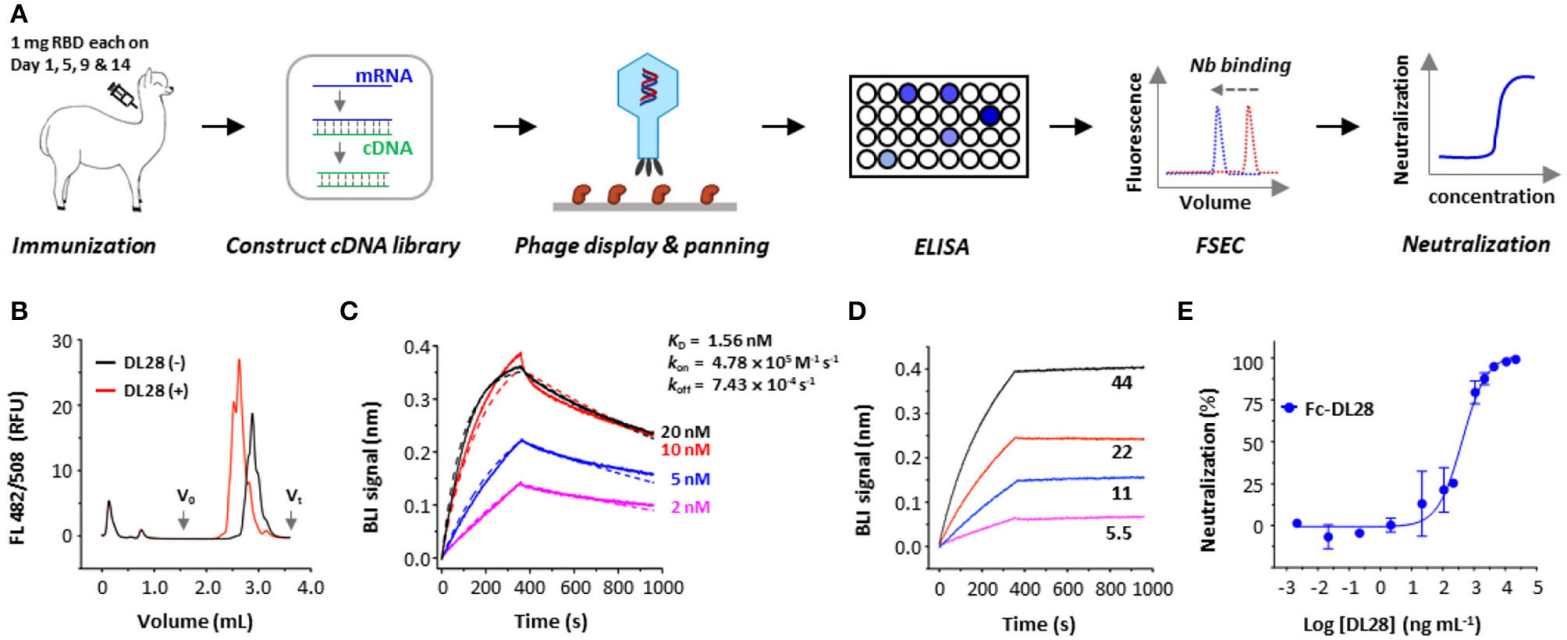
Strategy and isolation of neutralizing nanobodies. **(A)** Schematic flowchart for the identification of neutralizing nanobodies (Nbs). Immunization dose and schedule are indicated. The mRNA was isolated from an alpaca that was immunized with the RBD. A phage display library expressing nanobodies was selected against RBD. Positive clones were screened using ELISA and fluorescence-detector size exclusion chromatography (FSEC) for RBD-binding, and purified nanobodies were screened using neutralization assays with SARS-CoV-2 pseudoviruses. **(B)** Unpurified DL28 causes earlier elution of the fluorescently labeled RBD on analytic gel filtration. **(C)** Binding kinetics of DL28 to RBD using BLI with RBD immobilized and DL28 as analyte at indicated concentrations (nM). Solid lines indicate original data and dotted lines indicate fitted curves. **(D)** Evidence for the binding between DL28 and S protein. Apparent binding kinetics are not fitted due to the existence of bridged complexes between immobilized DL28 and the trimeric analyte S. **(E)** Neutralization assay of Fc-DL28 against SARS-CoV-2 pseudoviruses.

To elicit neutralizing nanobodies against SARS-CoV-2, an adult female alpaca was immunized four times using recombinantly expressed RBD. The enzyme-linked immunosorbent assay (ELISA) test of sera showed an antibody titer of ~1 × 10^6^ after four rounds of immunization compared with the pre-immunization sample. The mRNA isolated from the peripheral blood lymphocytes of RBD-immunized alpaca was reverse-transcripted into cDNA for the construction of a phage display library (Figure 1A). Three rounds of solution panning were performed with increasingly stringent conditions and an off-selection step to screen high-affinity nanobodies. Subsequent screening using ELISA and fluorescence-detection size exclusion chromatography (FSEC; Li et al., 2021a,b) identified binders with ELISA signal that is at least three times higher than a control nanobody, as well as the ability to shift the gel filtration peak of fluorescently labeled RBD at 0.5 μM (Figure 1A). We identified 28 unique clones as positive clones and we focus on DL28 for this study.

As shown in Figure 1B, the DL28 causes an earlier elution of RBD in FSEC. Using the biolayer interferometry (BLI) assay, we determined the binding affinity of DL28 with RBD (*K*_D_ = 1.56 nM) (Figure 1C) and demonstrated its ability to bind S (Figure 1D). Subsequent assays using SARS-CoV-2 pseudotyped particles bearing the S from the first-reported strain from Wuhan (termed the wild-type, WT) displayed an IC_50_ of 0.41 μg mL^−1^ (Figure 1E) for the Fc version of DL28 (Fc-DL28).

### DL28 Is Broadly Active Against SARS-CoV-2 VOCs

**Figure 2:**
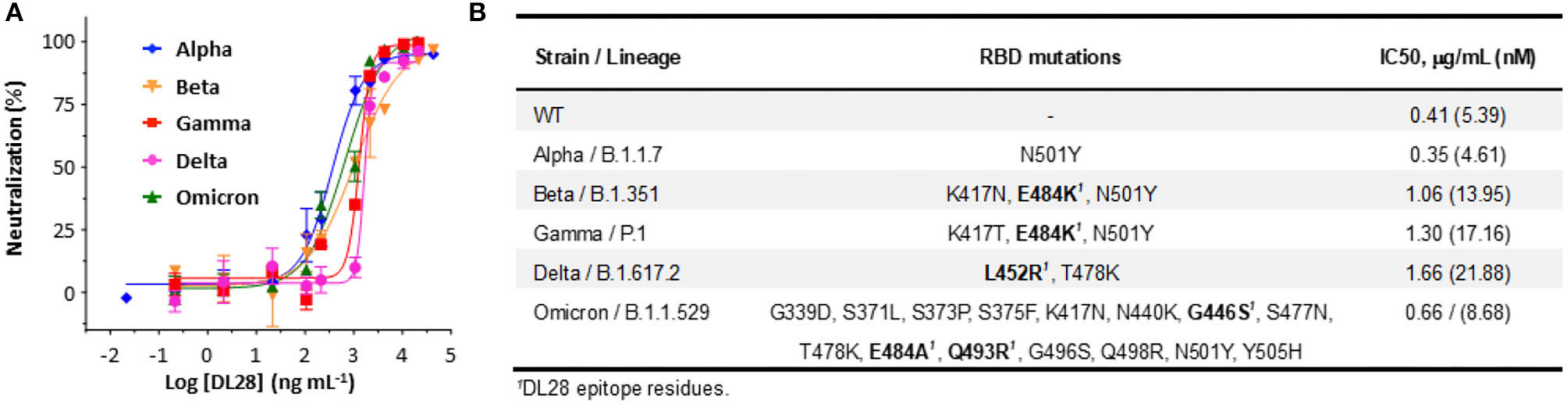
DL28 displays broad activity against SARS-CoV-2 variants. **(A)** IC_50_ determination. **(B)** A summary of variant information, RBD mutations, and IC_50_ values.

The destructive spread of VOCs, the Omicron variant in particular, poses new challenges to the public health systems. One of the central problems is the break-through infection by VOCs which essentially concerns the tolerance of antibodies to S mutations (mostly RBD mutations). To test whether DL28 possesses a broad-spectrum activity, we constructed SARS-CoV-2 pseudoviruses bearing the S from five VOCs, namely the Alpha (B.1.1.17), Beta (B.1.351), Gamma (P.1), Delta (B.1.617.2), and Omicron (B.1.1.529). The results showed that the mutations relating to the Alpha strain (N501Y, only RBD mutations are listed hereafter) did not affect the neutralizing activity of DL28, reporting a slightly lower IC_50_ (0.35 μg mL^−1^). The IC_50_ for the Beta (K417N/E484K/N501Y), Gamma (K417T/E484K/N501Y), and Delta (L452R/T478K) increased to 2.5, 3, and 4-fold, respectively (Figure 2A). Interestingly, despite having the highest number of mutations (Walter et al., 2022; Figure 2B), the Omicron strain remained sensitive to DL28 (IC_50_ = 0.66 μg mL^−1^; Figure 2A).

### Structural Interpretation of the Varying Activity of DL28 Against VOCs

To accurately characterize the epitope of DL28, we crystallized the DL28-RBD complex in the space group of *P*6_5_22 and solved its structure to 3.0-Å resolution by molecular replacement using published RBD and nanobody structures (Li et al., 2021a) as search models. The structure was refined to *R*_work_/*R*_free_ of 0.2264 / 0.2476 with no geometry violations (Table 1). The asymmetric unit contains two DL28-RBD complexes with high similarity (Cα RMSD of 0.51 Å). The chains A/C are used for structure description.

**Table 1:** Data collection and refinement statistics.

	**DL28-RBD**
**Data collection**	
Space group	P 6_5_ 2 2
Cell dimensions	
*a, b, c* (Å)	177.46, 177.46, 133.13
*α, β, γ* (°)	90, 90, 120
Wavelength (Å)	0.9792
Resolution (Å)	50.00 – 3.00 (3.11- 3.00)^a^
*R*_merge_	0.174 (1.565)
*R*_pim_	0.057 (0.509)
*I*/σ*I*	14.1 (1.3)
Completeness (%)	100.0 (100.0)
Multiplicity	9.2 (9.7)
*CC* ^*^ ^b^	0.997 (0.852)
**Refinement**	
Resolution (Å)	44.37 – 3.00
No. reflections	25,257
*R*_work_ / *R*_free_	0.2264 / 0.2476
No. atoms	5,026
Protein	4,878
Ligands	127
Solvent	21
No. residues	626
B-factors (Å^2^)	97.7
Protein	96.3
Ligand/ion	159.5
Solvent	60.8
R.m.s deviations	
Bond lengths (Å)	0.010
Bond angles (°)	1.582
Ramachandran	
Favoured (%)	97.09
Allowed (%)	2.91
Outlier (%)	0
**PDB ID**	7F5H

a *Highest resolution shell is shown in parenthesis*.

b CC${}^{*}=\sqrt{\frac{{2CC}_{\frac{1}{2}}}{1+CC_{\frac{1}{2}}}}$.

**Figure 3:**
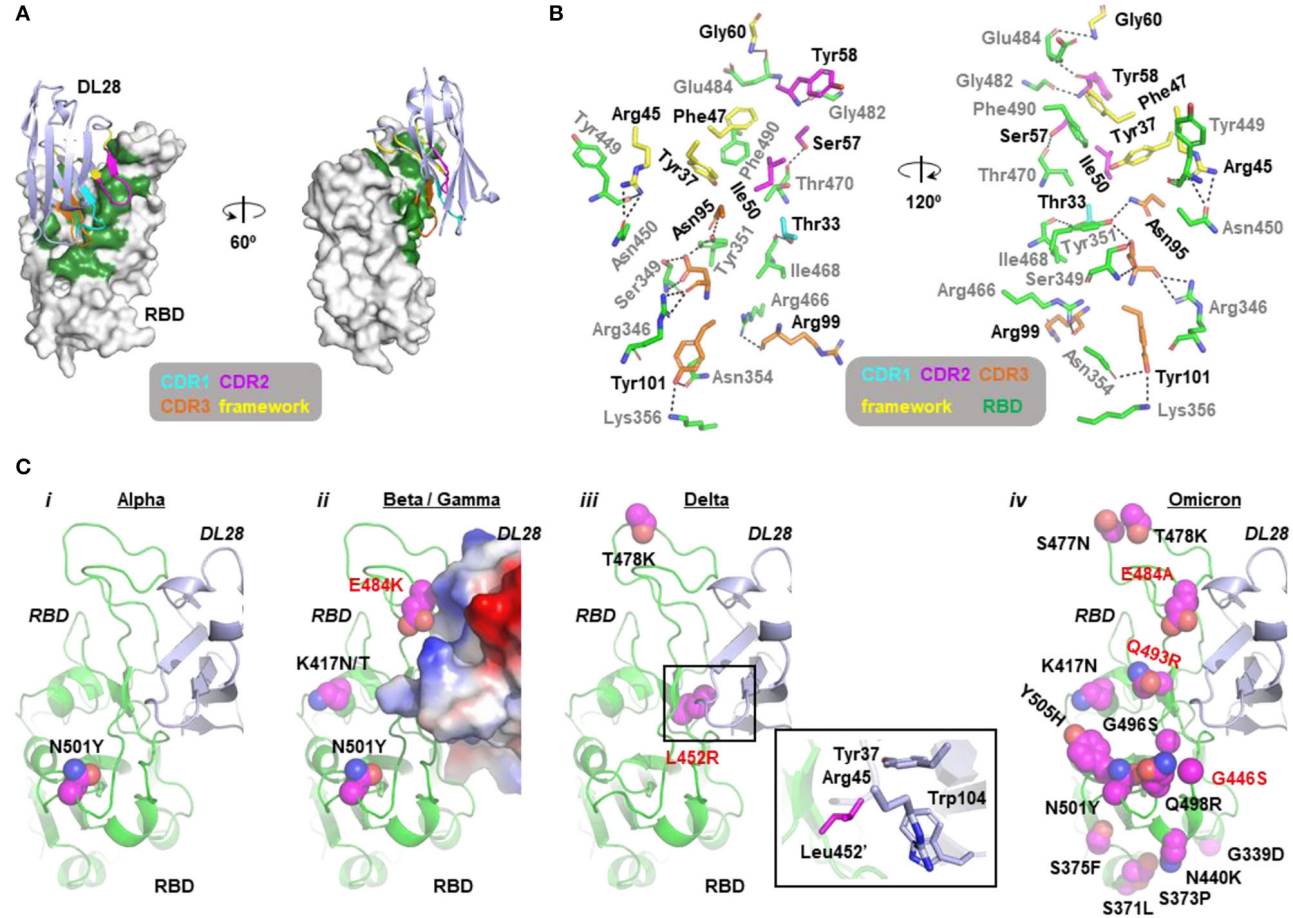
Molecular insights into the activity of DL28 against SARS-CoV-2 variants. **(A)** The overall structure of DL28 (light blue) in complex with RBD (white). DL28 binds the high-chair-shaped RBD at one side. The binding interface is colored green. Three complementarity-determining regions (CDRs) and the framework residues involved in the binding are color-coded as indicated. **(B)** Stick representation of the interaction residues from DL28 (cyan, magenta, orange, and yellow) and RBD (green). DL28 residues are labeled in black and RBD residues are labeled in gray. Dash lines indicate distances within 3.8 Å. **(C)** The distribution of RBD mutations (magenta sphere) from the Alpha *(i)*, Beta/Gamma *(ii)*, Delta *(iii)*, and Omicron *(iv)* variants in the context of the DL28 epitope. RBD (green) and DL28 (blue) are shown as ribbon representations except that DL28 is shown as Adaptive Poisson-Boltzmann Solver electrostatic potential surfaces in *ii*. The expanded view in *iii* highlights the interaction between Leu452' and indicated DL28 residues.

The RBD structure assembles a high-chair shape and DL28 binds to RBD at one side of the high-chair with a buried surface area (Krissinel and Henrick, 2007) of 986.3 Å^2^ (Figure 3A), with contributions of 41.9 Å^2^ from CDR1, 195.4 Å^2^ from CDR2, 377.8 Å^2^ from CDR3. The framework region also contributed significantly to the binding, with a buried surface of 371.2 Å^2^ (~40% of the total). Characteristically, most interactions are contained in CDR3 and only one residue in CDR1 is involved in the binding (Figure 3B). Overall, the interaction involves hydrophobic interactions, 17 hydrogen bonds, and a π-π interaction between Phe47 and Phe490' (Figure 3B, Supplementary Table 1; for clarity, we label residues from RBD with a prime).

Analysis of the crystal packing shows that regions around the RBD-DL28 interaction interface are involved in crystal contact mediated by a DL28 and an RBD molecule from two different adjacent asymmetric units (Supplementary Figure 1A). Despite having these two distinct packing patterns (Supplementary Figures 1B,C), the two copies of DL28-RBD complex showed superimposable conformations (Supplementary Figure 1D). This suggests that the observed interactions are unlikely to be influenced by crystal packing and hence are of functional significance.

The structural information offers insights into the broad activity of DL28 against SARS-CoV-2 variants. Consistent with the similar reactivity of DL28 against the original strain (WT) and the Alpha variant (Figure 2), the mutation from the Alpha strain (N501Y) is not involved in DL28-binding. For the three RBD mutations from the Beta/Gamma strain, K417N/T and the abovementioned N501Y are expected to be neutral because they are remote from the DL28 epitope. In contrast, E484K happens at a site adjacent to the DL28 epitope. Although the side chain of Glu484' was not involved in the DL28-binding (Figure 3B), the charge reversal by E484K would cause charge–charge repulsion with DL28 (Figure 3C). This may explain the mild resistance of the Beta/Gamma to DL28 (Figure 2). Similarly, although the T478K mutation from the Delta strain is distant from the DL28 epitope, the L452R mutation would weaken the interactions by diminishing hydrophobic interactions with Tyr37/Trp104 in the DL28 framework and introducing a charge–charge repulsion with Arg45. Finally, although the mutation spectrum in the Omicron overlaps with the epitope of DL28 by three residues (G446S/E484A/Q493R), the neutralizing activity of DL28 was not drastically altered. This will be discussed in the next section.

Consistent with its ability to bind to S (Figure 1D), the structure alignment of DL28-RBD with S reveals no clashes when DL28 is aligned onto the “up”-RBD, and only minor clashes with the NTD from the clockwise subunit when DL28 is aligned onto the “down”-RBD (Supplementary Figure 2). Whether and how DL28 binds “down”-RBDs in the context of S trimer remains to be experimentally determined.

### DL28 Unlikely Uses Direct Competition or Steric Hindrance to Block ACE2

**Figure 4:**
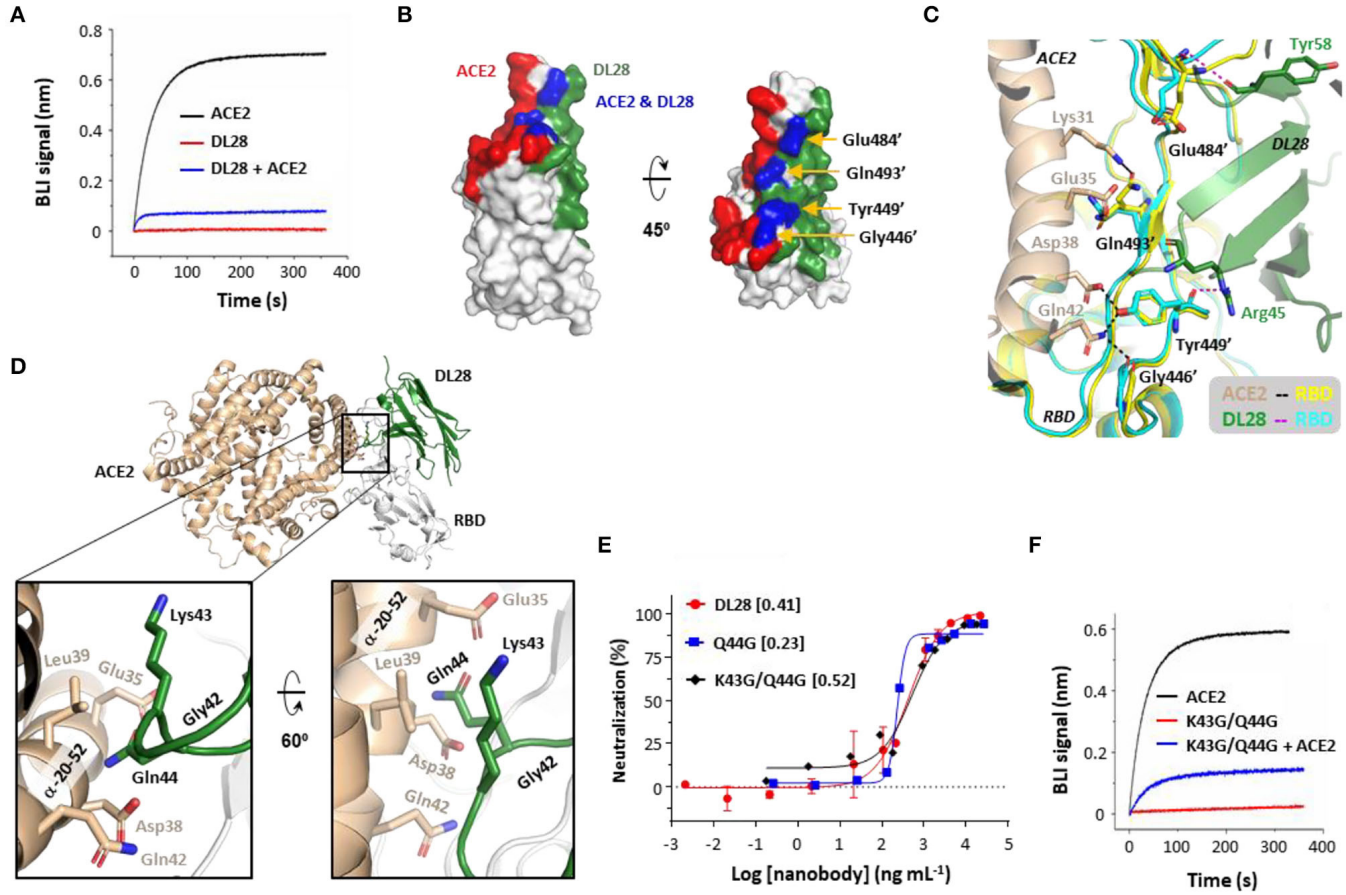
The ACE2-blocking activity of DL28 unlikely involves direct competition or steric hindrance. **(A)** Pre-incubation of DL28 with RBD blocks ACE2-binding. A sensor coated with RBD was first treated with 100 nM of DL28 before being incubated with a DL28-containing solution with (blue) or without (red) ACE2. As a control, the ACE2-RBD binding profile (black) was recorded without DL28 on a biolayer interferometry (BLI) system. **(B,C)** The overlap (blue) between the DL28 epitope (green) and the ACE2-binding site (RBM, red) **(B)** is speculated to be compatible for binding with both DL28 and ACE2 **(C)**. Black/magenta dashed lines indicate ACE2-RBD and DL28-RBD interactions, respectively. **(D–F)** The minor clashes between DL28 and ACE2 do not play a major role in cross-competition. **(D)** Gln44 on DL28 is in close contact with the RBD-interacting α-helix from ACE2 when the DL28-RBD structure is aligned onto the ACE2-RBD structure. **(E)** Neutralization assays for Q44G and K43G/Q44G using the SARS-CoV-2 WT strain. The data for DL28 are obtained from Figure 1E for comparison reasons. **(F)** The triple-glycine DL28 (Gly42, K43G/Q44G) retained the ability to inhibit ACE2 for RBD-binding. The experimental setting was the same as in **(A)**. Monovalent DL28 was used in **(A)** and Fc-dimers were used in **(E,F)**.

To probe neutralization mechanisms for DL28, we performed cross-competition binding assays and found that DL28 blocked receptor-binding to near completion (Figure 4A). Direct competition and steric hindrance are the two most common mechanisms for ACE2-blocking activity of antibodies. However, as analyzed below, DL28 does not seem to fall into either of the two categories.

For direct competition, the DL28 epitope and the RBM overlap by four residues, namely Gly446', Tyr449', Glu484', and Gln493' (Figure 4B). However, the overlap appears to be compatible with binding to both ACE2 and DL28 owing to their distinct interaction modes (Figure 4C). RBD Gly446' and Gln493' are only in proximity and do not form hydrogen bonds with DL28 (Supplementary Table 1); although ACE2 Gln42 forms a hydrogen bond with Gly446', and ACE2 Lys31 and Glu35 interact with the side-chain of Gln493', they approach RBD at the opposite of DL28 (Figure 4C). The rest of the two residues, Tyr449' and Glu484' form hydrogen bonds with DL28 *via* their main-chain atoms. The side chain of Tyr449' forms a hydrogen bond with ACE2 Asp38 and Gln42, but Glu484' only interacts with ACE2 *via* Van der Waals forces. Finally, both the main-chain and side chain of the four RBD residues showed negligible differences between the ACE2- and DL28-bound forms (Figure 4C). The analyses suggest a lack of direct competition between ACE2 and DL28. In line with this, simultaneous mutation of three of the four residues (G446S/E484K/Q493R), as found naturally in the Omicron strain (Figure 3C), did not cause appreciable resistance to DL28 (Figure 2).

For steric hindrance, aligning the DL28-RBD structure to the ACE2-RBD (Lan et al., 2020) structure revealed minor clashes between DL28 Lys43/Gln44 in a framework loop and the ACE2 α-helix α_20−52_ (subscript numbers refer to the start-end residues) (Figure 4D) which contains most of the key receptor–RBD interactions (Lan et al., 2020). To investigate if steric hindrance plays a role, we made a Q44G mutant to eliminate the side chain which protrudes to ACE2 α_20−52_ near Asp38 in the aligned model. Interestingly, the Q44G showed a slightly increased neutralizing activity (Figure 4E), excluding Gln44 as an ACE2-clashing residue. Further mutation of the adjacent Lys43 to glycine slightly reduced the ACE2-blocking (Figure 4F) and neutralization activity (Figure 4E). This result may be interpreted as Lys43 being a clashing residue. However, since the Lys43 side-chain points away from ACE2 in the aligned model, we favor an alternative possibility: the tri-glycine motif (together with Gly42) introduces structural instability to the nanobody framework and affects the orientation of the CDRs for tight binding. Taken together, we conclude that DL28 does not rely on direct competition or steric hindrance for the blocking of ACE2-binding. Rather, we propose a “conformation competition” mechanism for the neutralizing activity of DL28 (refer to Discussion).

## Discussion

SARS-CoV-2 S-RBD has been a focus for antibody development since the beginning of the outbreak. So far, there are more than 30 RBD-targeting nanobodies with their epitopes structurally characterized (Supplementary Figure 3). These RBD-targeting neutralizing nanobodies can be categorized into four classes (Class A–D) based on their mechanisms. The most common class (Class A) blocks ACE2-binding by direct competition (Custódio et al., 2020; Xiang et al., 2020; Ahmad et al., 2021; Güttler et al., 2021; Huo et al., 2021; Koenig et al., 2021; Li et al., 2021a; Pymm et al., 2021; Sun et al., 2021; Wagner et al., 2021; Walter et al., 2022) and their epitopes overlap with RBM (Supplementary Figure 3A). Class B nanobodies are also frequently reported and they impede ACE2-binding by steric hindrance (Supplementary Figure 3B; Hanke et al., 2020; Wrapp et al., 2020b; Güttler et al., 2021; Koenig et al., 2021; Pymm et al., 2021; Sun et al., 2021; Wagner et al., 2021; Xu et al., 2021; Walter et al., 2022). Class C nanobodies destabilize the S trimer (Huo et al., 2021; Sun et al., 2021) by targeting epitopes that are buried between RBD and the N-terminal domain of adjacent subunits (Supplementary Figure 3C). Finally, Class D nanobodies, represented by Nb6 from a synthetic library and C5 from immunized llama (Schoof et al., 2020; Huo et al., 2021), prevent ACE2-engagement by binding two RBDs and by locking the RBDs in the “down” conformation (Supplementary Figure 3D).

Although DL28 can block ACE2-binding, our results suggest that DL28 is unlikely a Class A/B nanobody. Rather, structural analysis, as will be described below, suggests a “conformation competition” mechanism through which DL28 keeps the “backrest” part of the RBD in a state incompatible with ACE2-binding.

**Figure 5:**
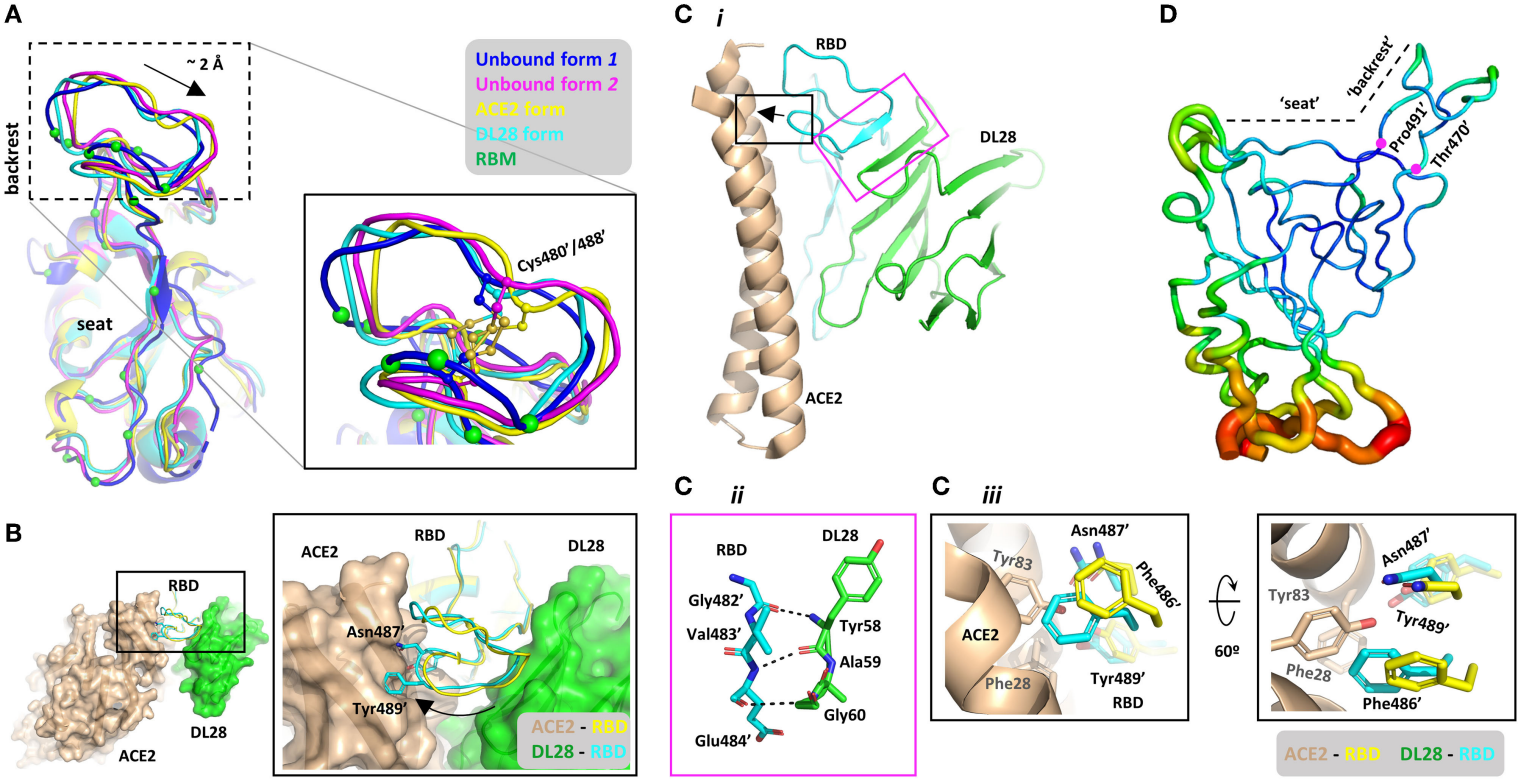
Structural basis for the proposed “conformation competition” mechanism for the ACE2-blocking activity of DL28. **(A)** Comparison of the RBD conformations at the RBM between two unbound forms (blue, magenta), the ACE2-bound (Lan et al., 2020) form (yellow), and the DL28-bound form (cyan). The ACE2-interacting residues are shown as green Cα spheres. **(B)** Alignment of the DL28-RBD structure (green surface and cyan ribbon) with the ACE2-RBD structure (Lan et al., 2020; wheat surface and yellow ribbon). DL28 pushes the boxed loop in **(A)** toward ACE2, causing clashes between two aromatic residues and the RBD-interacting α-helices in ACE2. **(C)** Both ACE2 and DL28 use a rigid structure to interact with the boxed loop in **(A)**, making a compromise unlikely to reach. The black box highlights the clash between ACE2 (wheat) and the DL28-bound form of RBD (cyan). The clashing RBD residues in the ACE2-bound form are shown as yellow sticks. The magenta box highlights the interaction between DL28 and the “backrest” region mediated by main-chain interactions. **(D)** Cα b-factor distribution shown in putty representation using a rainbow ribbon with a radius that increases from the lowest (61.6 Å^2^; dark blue) to the highest (170.0 Å^2^; red) B-factor. The average B-factor of the “backrest” region (residue 470–491) is 76.59 Å^2^ which is lower than that of the whole chain (97.5 Å^2^), suggesting relative inflexibility.

The “backrest” part of the RBM shows conformational dynamics (Figure 5A) in the unbound form (Zhou et al., 2020a). The two conformations with the most dramatic differences (dubbed Unbound form *1* and *2*) show a ~2.0-Å displacement. The DL28-bound form assumes a conformation more similar to Unbound form *1* while the ACE2-bound form assumes a similar conformation to Unbound form *2*. Superposing the ACE2-RBD onto the DL28-RBD structure reveals severe clashes between ACE2 α_20−52_ (numbers indicate start and end of second structures) and three RBM residues (Phe486', Asn487', Tyr489') in the DL28-bound form (black box, Figures 5B,C). Further, despite the conformation dynamics of the unbound RBD at the “backrest” and “seat” regions (Figure 5A and ref. Williams et al., 2022), they are relatively inflexible once bound with DL28 (Figure 5D) according to the b-factor analysis. Thus, unless ACE2 and DL28 make a compromise on the relative position to RBD, or if the “backrest” region assumes a compact conformation to accommodate both ACE2 and DL28, the receptor- and nanobody-binding events would be mutually exclusive.

But such a compromise is unlikely to reach. The transition from the DL28-bound form to the ACE2-bound form may be difficult because the “backrest” part is pushed by a 4-residue β-strand (β_56−59_) which is part of the stable DL28 framework consisting of four stacking β-strands (Figure 5C). In fact, DL28 β_56−59_ is aligned with the “backrest” loop such that a fragment within the loop (Gly482-Val483-Glu484) is transformed into a β-strand that stacks with the nanobody β-sheet (the magenta box, Figure 5C). Similarly, ACE2 also interacts with this “backrest” region *via* a rigid helix (α_20−52_) which lies on the top of RBM like a lever. A 2-Å displacement at the “backrest” area would push this lever at the N-terminal end (black arrow, Figure 5C) and cause the C-terminal end to disengage from the “seat” region unless the helix can deform/break. However, α-helices are generally rigid and α_20−52_ contains no helix-destabilizing residues, such as proline and glycine. Finally, the “backrest” region of RBD contains a disulfide bond made of Cys480'/488' (Figure 5A). This bridged structure endows the region with local rigidity, making it difficult for the backrest to be “compressed” by ACE2 and DL28. Based on the structural analyses, we propose that DL28 neutralizes SARS-CoV-2 by a “conformation competition” mechanism. Such a mechanism has not yet been reported in SARS-CoV-2 antibodies but a similar mechanism has been proposed for an antibody (named MERS-4) against the Middle East respiratory syndrome coronavirus (MERS-CoV; Zhang et al., 2018).

Notably, the epitope of DL28 overlaps with two Class C nanobodies, NB17, and Nb36 (Supplementary Figure 3C). Apart from this similarity, the “backrest” region of RBD in both Nb17-bound (Supplementary Figure 4A) and Nb36-bound form (Supplementary Figure 4B) also assumes a conformation that is incompatible with ACE2-binding, as observed for DL28 (Figure 5A). Despite this, these nanobodies cause no inhibition (Nb17) or only weak inhibition (Nb36) on ACE2-binding (Sun et al., 2021). This may reflect the structural differences between NB17/Nb36 and DL28. As shown in Supplementary Figure 4C, the “backrest” region in the Nb17-bound form unwinds into a flexible loop with the Cys480'-Cys488' disulfide bond being broken (we would note the possibility of model inaccuracy owing to the relatively low resolution (3.73 Å) of the reported structure). Thus, this flexible loop may swing away to avoid clashing with ACE2. In the case of Nb36, the nanobody is remote from the “backrest” region, leaving enough space for this region to assume a conformation compatible with ACE2-binding (Supplementary Figure 4D). By contrast, the “backrest” region binds tightly to DL28 (magenta box, Figure 5C) and the rigidity of the DL28 core would lock the loop in the current position.

Due to high demands and limited BSL3 laboratory resources during the continuing outbreak, the neutralization activity of DL28 against the authentic SARS-CoV-2 and the VOCs were not tested in this study. However, accumulating evidence from publications in this field since early 2020 has highlighted a strong correlation between assays using pseudovirus and authentic virus (Bewley et al., 2021; Jones et al., 2021; Li et al., 2021a,b). Thus, it is likely that DL28 would neutralize the authentic viruses too. Another immediate concern is the protection efficacy of DL28 in animal models. Previously, we have demonstrated that nanobodies, when in divalent forms, can protect hamsters from viral infection and lift symptoms (Li et al., 2021a). This observation was corroborated by several other studies (Huo et al., 2021; Nambulli et al., 2021; Pymm et al., 2021; Ye et al., 2021; Li et al., 2022). Such previous results warrant a more thorough investigation of the therapeutic potential of DL28 in the future.

We would note the modest neutralizing of DL28. As revealed by the binding competition assay, the block of ACE2-binding was incomplete. This may have caused the observed moderate neutralizing activity. Alternatively, the relatively low activity of DL28 may be an intrinsic feature for non-RBM-targeting antibodies. According to an antibody survey (Dejnirattisai et al., 2021), antibodies that target RBM generally neutralize SARS-CoV-2 with higher activity, and the exact reasons are yet to be discovered. Nevertheless, non-RBM-targeting antibodies like DL28 are attractive candidates for therapeutic cocktails due to their generally broader spectra (Huo et al., 2020b; Pinto et al., 2020; Zhou et al., 2020b; Li et al., 2021b).

In summary, we report a nanobody with neutralizing activity against SARS-CoV-2 VOCs including the Omicron. Structural characterizations rationalize the tolerance of DL28 for mutations found in various VOCs. Mutagenesis, binding assays, and structural analyses suggest a “conformation competition” mechanism through which DL28 locks RBD in a state incompatible for receptor engagement. Since the epitope of DL28 only marginally overlaps with the RBM, DL28 may be able to bind to RBD in the presence of other RBM-targeting nanobodies and human monoclonal antibodies. Such pairs will allow for the development of biparatopic nanobodies to increase the tolerance of escape mutants and the high affinity of DL28 could offer great advantages in such applications.

## Materials and Methods

### Protein Expression and Purification—Spike (S)

The polypeptide containing, from N- to C-terminus, residues Met1 – Gln1208 (without the C-terminal transmembrane helix, Uniprot P0DTC2) of the SARS-CoV-2 S with mutations K986P/V987P, a GSAS linker substituting the furin sites (Arg682-Arg685), a C-terminal T4 fibritin trimerization motif (GYIPEAPRDGQAYVRKDGEWVLLSTFL), a tobacco etch virus (TEV) protease cleavage site, a FLAG tag and a polyhistidine tag (Zhang et al., 2021) was encoded in a pCDNA3.1 backbone vector and overexpressed in Expi293 cells by transient transfection using polyethylenimine (PEI). After 3.5 days of suspension culturing, the medium was harvested by filtration through a 0.22-μm membrane, and adjusted to contain 200 mM of NaCl, 20 mM of imidazole, 4 mM of MgCl_2_, and 20 mM of Tris-HCl pH 7.5. The filtrate was incubated with 3 mL of Ni-NTA beads at 4°C for 2 h. The beads were loaded into a Bio-Rad gravity column, washed with 50 column volume (CV) of 20 mM of imidazole, and subsequently eluted with 250 mM of imidazole in 200 mM of NaCl, and 20 mM of Tris-HCl pH 7.5. Fractions containing S were pooled, concentrated with a 100-kDa cut-off membrane concentrator, and further purified by gel filtration. The S protein was quantified using a theoretical ε_280_ of 138,825 M^−1^ cm^−1^.

### Protein Expression and Purification—RBD

The polypeptide containing, from N- to C-terminus, the honey bee melittin signal peptide (KFLVNVALVFMVVYISYIYAA), a Gly-Ser linker, residues 330-531 of the SARS-CoV-2 S (Uniprot P0DTC2), a Gly-Thr linker, the 3C protease site (LEVLFQGP), a Gly-Ser linker, the Avi tag (GLNDIFEAQKIEWHE), a Ser-Gly linker, and a deca-His tag were encoded in a pFastBac-backbone vector for overexpression in *Trichoplusia ni* High Five suspension cells. Cells at 2 × 10^6^ cells per milliliter were transfected with baculovirus generated using standard Bac-to-Bac procedures (Invitrogen) and the expression was allowed for 48–60 h at 27°C in flasks. The medium from 1 L of culture was filtered using a 0.22-μm membrane and the filtrate was adjusted to contain 30 mM of imidazole before incubating with 3.0 mL of Ni-Sepharose Excel (Cat. 17-3712-03, GE Healthcare) beads for 2 h at 4°C with mild agitation. The beads were loaded into a gravity column, washed with 10 CV of 20 mM of imidazole, and eluted using 300 mM of imidazole in 150 mM of NaCl and 20 mM of Tris HCl pH 8.0. For site-specific biotinylation, the Avi-tagged RBD at 0.8 mg mL^−1^ was incubated with 5 mM of ATP, 10 mM of magnesium acetate, 43.5 μM of biotin, 22 μg mL^−1^ of home-purified BirA in a 3.2-mL reaction mix and incubated at 4°C for 16 h. Biotinylated RBD was concentrated with a 10-kDa cut-off membrane to ~3 mg mL^−1^ before loaded onto a Superdex Increase 200 10/300 GL column for gel filtration. Fractions containing the RBD were pooled, aliquoted, flash-frozen in liquid nitrogen, and stored at −80°C before use.

For crystallization, the RBD eluted from the Ni-NTA column was desalted using a desalting column and digested with home-purified 3C protease to remove the C-terminal tags. The resulted tag-free RBD was mixed with nanobodies (refer to the section below) at a molar ratio of 1:1.3 and the mix was loaded onto a Superdex Increase 200 10/300 GL column for gel filtration. Fractions containing the complex were pooled and concentrated to 10 mg mL^−1^ for crystallization.

### Protein Expression and Purification—Monovalent Nanobodies in *Escherichia coli*

Monovalent nanobodies were expressed with a C-terminally Myc tag and a hexahistidine tag in *Escherichia coli* (*E. coli*) MC1061 cells. Briefly, cells carrying nanobody-encoding pSb-init plasmids (Zimmermann et al., 2018) were grown in Terrific Broth (TB, 0.017 M of KH_2_PO_4_ and 0.072 M of K_2_HPO_4_, 1.2 %(w/v) of tryptone, 2.4 %(w/v) of yeast extract, 0.5% (v/v) glycerol) supplemented with 25 mg L^−1^ of chloramphenicol at 37°C with shaking at 200 rpm. When cell density reached an OD_600_ of 0.5 (~2 h), the shaker was set to 22°C and the cells were allowed to grow for another 1.5 h before added with 0.02% (w/v) arabinose for induction for 17 h. Cells were harvested by centrifugation and lysed by osmotic shock as follows. Briefly, cells from 1 L of culture were resuspended in 20 mL of TES-high Buffer (0.5 M sucrose, 0.5 mM EDTA, and 0.2 M Tris-HCl pH 8.0) and incubated at 4°C for 30 min. Dehydrated cells were then abruptly rehydrated using 40 mL of ice-cold MilliQ H_2_O at 4°C for 1 h to release periplasmic protein. The periplasmic extract was collected by centrifugation at 20,000 × *g* at 4°C for 30 min. The supernatant was adjusted to have 150 mM of NaCl, 2 mM of MgCl_2_, and 20 mM of imidazole before being incubated with Ni-NTA beads that had been pre-equilibrated with 20 mM of imidazole, 150 mM of NaCl, and 20 mM of Tris HCl pH 8.0. After batch-binding for 2 h, the Ni-NTA beads were washed using 30 mM of imidazole, before being eluted using 300 mM of imidazole, 150 mM of NaCl, and 20 mM of Tris HCl pH 8.0. Nanobodies were quantified using their theoretical molar extinction coefficient calculated based on the contents of aromatic residues.

### Protein Expression and Purification—Divalent Nanobodies in Mammalian Cells

Nanobodies with a C-terminal Fc fusion and an N-terminal leader peptide (MEFGLSWVFLVALLRGV) were transiently expressed in Expi293 suspension cells. Briefly, cells at 2.5 × 10^6^ cells per milliliter were transfected with a mix of plasmids and PEI. Valproic acid was included at 2 mM to increase the expression. After 65 h at 37°C, the medium was harvested by centrifugation at 1,000 × *g* and filtration. The filtrate was incubated with rProtein A beads (Cat. SA012005, SmartLifesciences, China) for batch binding at 4°C for 3 h. The beads were packed into a gravity column, washed using 20 CV of PBS buffer before being eluted using 0.1 M of glycine pH 3.0. The elution was immediately neutralized with 1 M of Tris HCl pH 8.0. The buffer was then exchanged to PBS on a Bio-Rad desalt column.

Nanobody mutants in this study were all generated on the Fc-fusion constructs using standard PCR-based site-directed mutagenesis protocols. DNA sequences were verified by sequencing, and the mutants were expressed and purified the same way as their wild-type proteins.

### Alpaca Immunization and Antibody Titer Determination

Purified RBD (0.5 mL at 2 mg mL^−1^) was mixed with an equal volume of the Gerbu adjuvant (Cat. 3111) by vortexing. The resulted emulsion was injected by the subcutaneous route at 10 sites near the bow lymph node in the neck base of an adult female alpaca (3-years old). The immunization process was repeated thrice (a total of four rounds) with 4 days between each injection.

To determine the antibody titer, 3 mL of blood samples before and after each injection were collected. After 2 h at room temperature (RT, 20–25°C), the clotted sample was centrifuged at 3,000 × *g* for 5 min at RT to collect the sera in the supernatant. Wells of 96-well plates (Maxisorp, Nunc Thermo Fisher Scientific) were coated overnight at 4°C with 100 μL of 2 μg mL^−1^ of biotinylated RBD in Tris-HCL buffer solution (TBS) (150 mM of NaCl, 20 mM of Tris-HCl, pH 8.0) and blocked with 0.5% of bovine serum albumin (BSA) in TBS. After washing five times with TBS, the serially diluted alpaca sera were added and incubated for 1 h. After washing, the bound nanobody was detected by HRP-conjugated Goat anti-Alpaca IgG (Cat. S001P, NBbiolab) using tetramethylbenzidine (TMB; Cat. T2885, Merck) as a substrate for horseradish peroxidase (HRP). The ELISA test of sera showed an antibody titer of ~1 × 10^6^ after four rounds of immunization compared with the pre-immunization sample.

### Phage Display Library Construction and Panning

Eighty milliliters of blood were collected from the immunized alpaca in EDTA-coated tubes. The tubes were inverted twice to inhibit coagulation. The peripheral blood lymphocytes were isolated using Ficoll Plus (density of 1.077 g mL^−1^) according to the manufacturer's instructions. Isolated lymphocytes were used for mRNA isolation with RNAsio Plus (TaKara). Reverse transcription was performed using mRNA and a commercial kit (Cat. R312-01, Vazyme). Polymerase chain reaction (PCR) was carried out with 50 ng of cDNA and the primer pair CALL001 (5'-GTCCTGGCTGCTCTTCTACAAGG-3') and CALL002 (5'-GGTACGTGCTGTTGAACTGTTCC-3') using the PCR Master Mix (Cat. 10149ES01, YEASEN Biotech, Shanghai, China). The PCR product was loaded onto a 1.5% (w/v) agarose gel and the 700-bp band was excised. The purified PCR product was used for the second round of PCR using the prime pair, VHH-BspQI-F (5'-ATAT*GC TCTTC*AAGTCAGGTGCAGCTGCAGGAGTCTGGRGGAGG-3') and VHH-BspQI-R (5'-TATA*GCTCTTC*CTGCCGAGGAGACGGTGACCTGGGT-3') which anneals to the framework 1 and framework 4 regions of nanobodies, respectively. The primers contained a recognition site (italic) for the Type IIs restriction enzyme, *BspQ*I for cloning purposes. The PCR product was purified using a FastPure kit (Cat. DC301, Vazyme).

One microgram of the PCR product and 10 μg of the pDX_init vector (Zimmermann et al., 2018) were digested separately with 50 units of *BspQ*I (Cat. R0712L, New England Biolabs) for 1.5 h at 50°C before heat inactivation at 80°C for 10 min. The digested DNA was gel-purified and 0.3 μg of the PCR product was mixed with 1.2 μg of vector and 10 units of T4 ligase in the ligation buffer (Cat. B110041, Sangon Biotech, Shanghai, China) for 1.5 h. The mixture was transformed into *E. coli* SS320 cells by electroporation in a 2-mm cuvette using a Gene Pulser Xcell (Bio-Rad) with a setting of 2,400 volts, 25 μF, and 750 Ω.

Cells were grown in 225 mL of 2-YT broth [1.0 %(w/v) yeast extract, 1.6 %(w/v) tryptone, 0.5 %(w/v) NaCl, pH 7.0] supplemented with 200 μg mL^−1^ of ampicillin and 2% (w/v) glucose in a 37°C shaking incubator at 220 rpm. To 10 mL of the overnight culture, 27 μL of the M13KO7 helper phage at 10^12^ plaque-forming units of mL^−1^ were added. After brief mixing, the mixture was incubated at 37°C for 30 min. The cells were collected by centrifugation at 3,200 × *g* for 10 min, resuspended in 2-YT broth supplemented with 200 μg mL^−1^ of ampicillin and 25 μg mL^−1^ of kanamycin, and placed in a shaker incubator at 37°C with 160 rpm.

After 16 h of culture, the medium from 50 mL of culture was collected by centrifugation at 3,200 × *g* for 30 min at 4°C. The supernatant (40 mL) was transferred to a fresh Falcon tube. Phage particles were precipitated by incubating the supernatant with 10 mL of 20%(w/v) PEG 6,000 and 2.5 M of NaCl for 30 min on ice. Precipitated phage particles were collected by centrifugation at 3,200 × *g* for 30 min at 4°C before being resuspended in 1 mL of PBS buffer. After centrifugation at 20,000 × *g* for 5 min, the supernatant was transferred into a fresh 1.5 mL tube and the procedure was repeated once.

The first round was performed in a Nunc Maxisorp 96-well immunoplate. The plate was first coated with 67 nM of neutravidin (Cat. 31000, Thermo Fisher Scientific) overnight at 4°C, followed by blocking with TBS buffer supplemented with 0.5 %(w/v) BSA for 30 min. Phage particles (4.9 mL) were incubated with 50 nM of biotinylated RBD, added to the neutravidin-coated wells, washed, and released from the plate by tryptic digestion (10 min at RT) with 0.25 mg mL^−1^ of trypsin in the buffer containing 150 mM of NaCl and 20 mM of Tris-HCl pH 7.4. After being treated with the trypsin inhibitor, AEBSF, the selected phage particles were amplified in *E. coli* SS320, and the second solution panning was performed as the first round except that the plate was replaced with 12 μL of MyOne Streptavidin C1 beads (Cat. 65001, Invitrogen). The bound-phage particles were challenged with 5 μM of non-biotinylated RBD to compete off binders with fast off-rates. The third round of panning was performed the same as the second round except that the RBD concentration was at 5 nM. The particles were eluted, and the phagemid was sub-cloned into pSb_init vector by fragment-exchange (FX) cloning and transformed into *E. coli* MC1061 cells for periplasmic expression and screening.

### ELISA—Nanobody Selection

Single colonies carrying pSb-init plasmids were grown at 37°C for 5 h in a shaking incubator at 300 rpm before 1:20 seeded into 1 mL of fresh TB supplemented with 25 μg mL^−1^ of chloramphenicol. Cells were induced with 0.02% (w/v) of arabinose at 22°C for 17 h before being collected by centrifugation at 3,000 × *g* for 30 min. Cell pellets were resuspended in TES Buffer [20 % (w/v) sucrose, 0.5 mM of EDTA, 0.5 μg/mL of lysozyme, 50 mM of Tris-HCl pH 8.0], and incubated for 30 min at room temperature (RT, 20–25°C). The lysate was added with 0.9 mL of TBS (150 mM NaCl, 20 mM of Tris-HCl pH 7.4) supplemented with 1 mM of MgCl_2_. The mix was centrifuged at 3,000 × *g* for 30 min at 4°C and the supernatant containing nanobodies was used for ELISA as follows.

Wells of a Maxi-Sorp plate (Cat. 442404, Thermo Fisher) was coated with Protein A at 4°C for 16 h. The plate was then blocked by 0.5 % (w/v) of BSA in TBS buffer for 30 min at RT and washed thrice using TBS before being incubated with anti-Myc antibodies at 1:2,000 dilution in TBS-BSA-T buffer [TBS supplemented with 0.5% (w/v) of BSA and 0.05 % (v/v) of Tween 20] for 20 min at RT. The plate was then washed thrice with TBST (TBS supplemented with 0.05% of Tween 20) to remove excess antibodies. The wells were incubated with the Myc-tagged nanobodies prepared over 20 min at RT. After washing thrice with TBST, the wells were incubated with 50 nM of biotinylated RBD or maltose-binding protein (MBP, as a control) for 20 min at RT. The wells were again washed thrice with TBST before being incubated with streptavidin-conjugated with horseradish peroxidase (HRP) (1:5,000, Cat S2438, Sigma). After 30 min, the plate was washed thrice with TBST. ELISA signal (absorbance at 650 nm) was developed by incubating the wells with 100 μL of developing reagents [51 mM of Na_2_HPO_4_, 24 mM of citric acid, 0.006% (v/v) of H_2_O_2_, 0.1 mg mL^−1^ 3,3',5,5'-tetramethylbenzidine] at RT.

### Fluorescence-Detection Size Exclusion Chromatography—Nanobody Selection

Fluorescence-detection size exclusion chromatography (FSEC) analysis of RBD-binding by nanobodies was performed as previously described (Li et al., 2021a). Biotinylated RBD was incubated with streptavidin (Cat. 16955, AAT Bioquest) which was chemically labeled by fluorescein. The fluorescent complex (500 nM) was mixed with the cell lysate containing unpurified nanobodies and the mixture was applied onto an analytic gel filtration column (Cat. 9F16206, Sepax) connected to an HPLC system equipped with a fluorescence detector (RF-20A, Shimadzu) for FSEC analysis. The FSEC profile was monitored by fluorescence at the excitation/emission pair of 482/508 nm and compared to that incubated with a control MBP-nanobody for peak shift.

### Biolayer Interferometry for S-Nanobody Binding and Competitive Binding

The binding kinetics was measured by a BLI assay using an Octet RED96 system (ForteBio). For DL28-RBD binding, biotinylated RBD (2 μg mL^−1^) was immobilized on an SA sensor by incubating with the sensor in the BLI Buffer [0.05 %(v/v) Tween 20, 1 × phosphate-buffered saline] at 30°C. The sensor was then placed in various concentrations (2, 5, 10, and 20 nM) of DL28 for 360 s (association). For dissociation, the sensors were moved into DL28-free BLI buffer, and the signal was monitored for 600 s. Data were fitted for a 1:1 stoichiometry for *K*_D_, *k*_on_, and *k*_off_ calculations using the built-in software Data Analysis 10.0.

For DL28-S binding, a streptavidin-coated SA sensor (Cat. 18-5019, Sartorius) was coated with 5 μg mL^−1^ of biotinylated nanobodies for ~1 min. The sensor was equilibrated in a nanobody-free buffer for ~30 s, before being bathed in solutions containing various concentrations (association) of Spike (analytes) for 360 s. For dissociation, the sensors were placed back into nanobody-free buffer. Binding kinetics were not fitted for DL28-S binding.

For competition between ACE2 and DL28, biotinylated RBD (2 μg mL^−1^) was immobilized on an SA sensor by incubating with the sensor in the BLI Buffer at 30°C. The RBD-loaded sensor was saturated in 100 nM of DL28 for 6–15 min. The sensor was then bathed in nanobody solutions with or without 100 nM of ACE2 (Cat. 10108-H08B, SinoBiological). The association of ACE2 was monitored for 360 s. As a control, the ACE2-RBD binding profile was recorded using the same procedure as above but in the absence of nanobodies.

### Crystallization

Crystallization trials were set up in a two-well sitting-drop plate with 70 μL of reservoir solution, and 1 μL each of the protein solution and the precipitant solution. The plates were incubated at 16°C for crystal growth. The precipitant solution contained 20% (w/v) of polyethylene glycol 3,350, and 0.2 M of potassium phosphate dibasic. Cryo protection was achieved by adding 20% (v/v) of glycerol in the respective precipitant condition. Crystals were harvested using a MitGen loop, and flash-cooled in liquid nitrogen before X-ray diffraction data collection.

### X-Ray Data Collection and Structure Determination

X-ray diffraction data were collected at beamline BL18U1 at Shanghai Synchrotron Radiation Facility with a 50 × 50 μm beam on a Pilatus detector at a distance of 450 mm, with oscillation of 0.5° and a wavelength of 0.97915 Å. Data were processed using HKL2000 (Otwinowski and Minor, 1997). The structure was solved by molecular replacement using Phaser (McCoy et al., 2004) with the individual RBD and nanobody structures (PDB 7C8W) as the search model. The model was built with 2*F*_o_-*F*_c_ maps in Coot (Emsley and Cowtan, 2004), and refined using Phenix (Adams et al., 2010). The structure was visualized using PyMol.

### Neutralization Assay Using SARS-CoV-2 Pseudoviruses

Retroviral pseudotyped particles were generated by co-transfection of HEK293T cells using PEI with the expression vectors encoding the various viral envelope glycoproteins, the murine leukemia virus core/packaging components (MLV Gag-Pol), and a retroviral transfer vector harboring the gene encoding the green fluorescent protein (GFP). The S protein expressed by phCMV-SARS-CoV-2 has been truncated to remove 19 amino acid residues at the C-terminal. Supernatants that contained pseudotyped particles were harvested 48 h post-transfection and filtered through a 0.45-μm membrane before neutralizing the assays.

VeroE6-hACE2 cells (10^4^ cells/well) were seeded into a 48-well plate and infected 24 h later with 100 μL of virus supernatant in a final volume of 150 μL. Nanobodies were pre-incubated with the pseudotype samples for 1 h at 37°C before cell/virus co-incubation. After 6 h of co-incubation, the supernatants were removed, and the cells were incubated in the medium for 72 h at 37°C. The GFP expression was determined by fluorescence-activated flow cytometry analysis (FACS). The infectivity of pseudotyped particles incubated with nanobodies was compared with the infectivity using pseudotyped particles and Dulbecco's modified Eagle's medium, 2% of fetal calf serum only and normalized to 100%.

SARS-CoV-2 pseudotypes for the Alpha (B.1.1.7), Beta (B.1.351), Gamma (P.1), Delta (B.1.617.2), and Omicron (B.1.1.529) variants were generated by incorporating the corresponding S mutations into the phCMV-SARS-CoV-2 plasmid. Desired mutations were verified by DNA sequencing. For the Omicron strain, the S protein contains the following mutations: A67V, del69-70, T95I, del142-144, Y145D, del211, L212I, insert214EPE, G339D, S371L, S373P, S375F, K417N, N440K, G446S, S477N, T478K, E484A, Q493R, G496S, Q498R, N501Y, Y505H, T547K, D614G, H655Y, N679K, P681H, N764K, D796Y, N856K, Q954H, N969K, and L981F. Since this first sequence (BA.1), other B.1.1.529 isolates such as BA.1.1 usually include an additional R346K mutation which is absent from our constructs in this study.

### Animal Experiment and Ethics

The alpaca immunization procedures were conducted in conformity with the institutional guidelines for the care and use of laboratory animals, and the protocols were approved by the Institutional Committee of Ethics and Research of the Central Laboratory at Xinyang Agricultural and Forestry University.

## Data Availability Statement

The structure factors and coordinates of the RBD-DL28 complex are available through the protein data bank (PDB) under accession code 7F5H.

## Ethics Statement

The animal study was reviewed and approved by the Institutional Committee of Ethics and Research of the Central Laboratory at Xinyang Agricultural and Forestry University.

## Author Contributions

TL and YLa selected, purified, and characterized nanobodies under the supervision of DLi, BZ, and SH performed neutralizing assays under the supervision of DLa, YZ, and SW immunized alpaca and constructed phage-display library. YZ performed crystallization and collected X-ray diffraction data under the supervision of JT, AG, and SB constructed S mutants and helped establish neutralization assays. YLi purified proteins and performed binding assays. JB helped with protein engineering. DLi and ZL processed X-ray diffraction data and solved structures. DLi analyzed structure, designed mutants, and wrote the manuscript with input from TL and DLa. All authors contributed to the article and approved the submitted version.

## Funding

This work has been supported by the National Natural Science Foundation of China (82151215 and 31870726, DLi; 31870153, DLa), the Strategic Priority Research Program of CAS (XDB37020204, DLi), Key Program of CAS Frontier Science (QYZDB-SSW-SMC037, DLi), the CAS Facility-based Open Research Program (2017, DLi), the National Key R&D Program of China (2020YFC0845900, DLa), the CAS president's international fellowship initiative (2020VBA0023, DLa), Science and Technology Commission of Shanghai Municipality (20ZR1466700, 22ZR1468300, DLi), and Shanghai Municipal Science and Technology Major Project (20431900402, DLa).

## Conflict of Interest

A patent application for potential nanobody therapy for the treatment of COVID-19 has been filed for DL28. YZ, SW, and JT were employed by the company Nanjing Crycision Biotech Co., Ltd., Nanjing, China. The remaining authors declare that the research was conducted in the absence of any commercial or financial relationships that could be construed as a potential conflict of interest.

## Publisher's Note

All claims expressed in this article are solely those of the authors and do not necessarily represent those of their affiliated organizations, or those of the publisher, the editors and the reviewers. Any product that may be evaluated in this article, or claim that may be made by its manufacturer, is not guaranteed or endorsed by the publisher.
