# Supplementary material for: Structural Characterization of a Neutralizing Nanobody With Broad Activity Against SARS-CoV-2 Variants
Source: Front Microbiol. 2022 Jun 2;13:875840. doi: 10.3389/fmicb.2022.875840 (PMC9201380; doi:10.3389/fmicb.2022.875840)
Supplement: Supplementary file 1 [file Data_Sheet_1.pdf]

Structural characterization of a neutralizing nanobody with broad activity against SARS-CoV-2 variants

Authors:

Tingting Li[1,#], Bingjie Zhou[2,3,#], Zhipu Luo[4,#], Yanling Lai[1], Suqiong Huang[2,3,5], Yuanze Zhou[6], Yaning Li[1], Anupriya Gautam[2,3], Salome Bourgeau[2,3,7], Shurui Wang[6], Juan Bao[1], Jingquan Tan[6], Dimitri Lavillette[3,8,*], Dianfan Li[1,*]

Supplementary Table 1

Supplementary Figures 1-4

Supplementary Table 1. A summary of interactions between DL28 and RBD.

DL28 Region	RBD	Interaction
CDR1		
[Ser31][a]	Ile468	VDW[b] force
Thr33	[Ile468]	H-bonding[b]
CDR2		
Ile50	Phe490	HPhI[b]
Ser52	[Ser469]	VDW force
Ser55	Glu471	VDW force
Thr56	[Gly482]	VDW force
Ser57	[Thr470]	H-bonding
[Tyr58]	[Gly482], [Glu484]	H-bonding
CDR3		
Asn95	Tyr351	H-bonding
Val97	Asp428	VDW force
[Asp98]	Arg466	VDW force
[Arg99]	Arg466	H-bonding
Trp100	[Arg355]	VDW force
Tyr101	Asn354, Lys356	H-bonding
(Asp102)[c]	Arg346, (Ser349), Tyr351	VDW force
Tyr103	Arg346	VDW force
Trp104	Asn450	VDW force
Framework		
Tyr37	Phe490, Leu495	HPhI
Gln39	Tyr449, [Gly446]	VDW force
Gln44	Tyr449	VDW force
Arg45	[Tyr449], Asn450	H-bonding
Arg45	Gln493	VDW force
Phe47	Phe490	π-π stacking
Ala59	[Gly482]	VDW force
[Gly60]	[Glu484]	H-bonding

[a]Brackets indicate interactions via mainchain. [b]Abbreviations: HPhI, hydrophobic interaction; VDW force, Van de Waals force; H-bonding, hydrogen bonding. [c]Parentheses indicate interactions through both sidechain and mainchain. Plain format indicates interactions via sidechain.

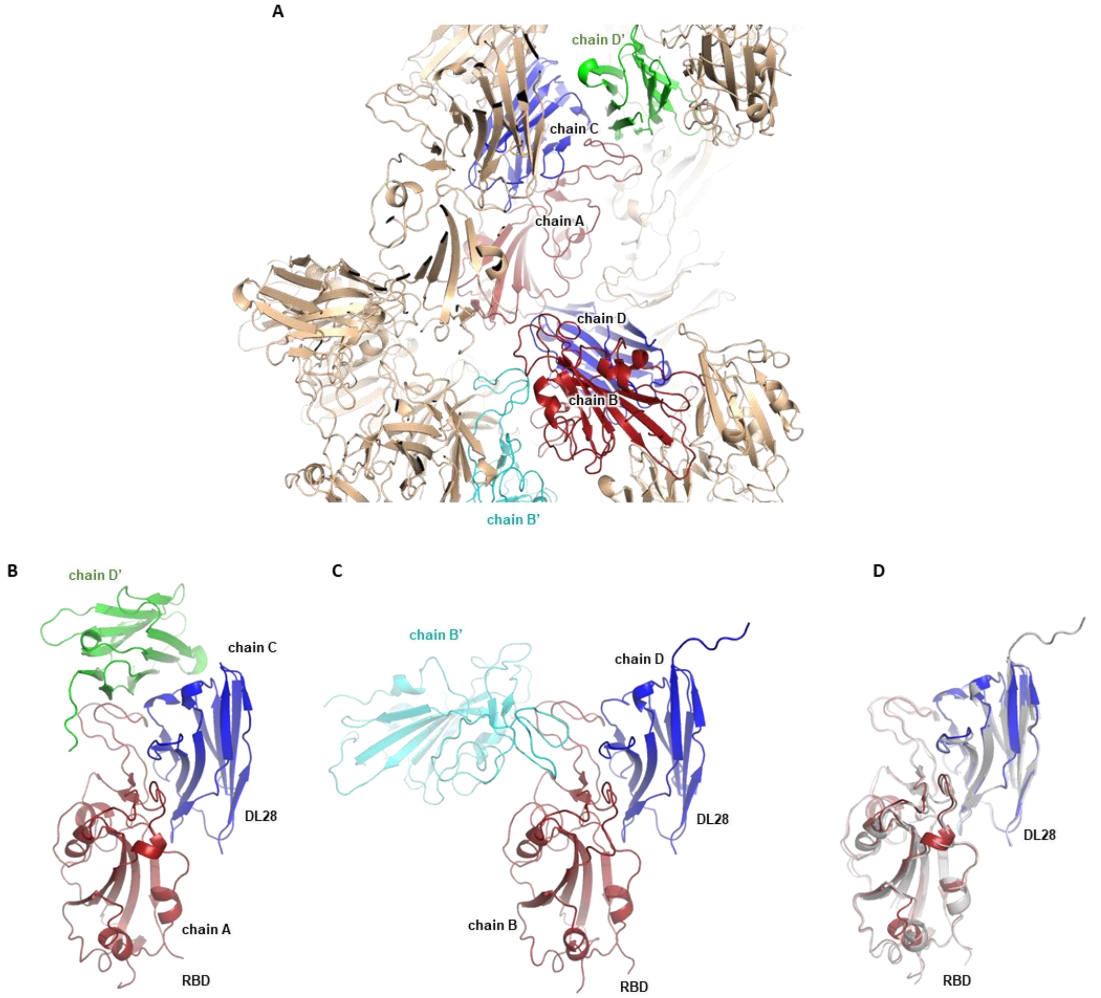

Supplementary Figure 1. The DL28-RBD interactions are unlikely influenced by crystal packing. (**A**) Overview of crystal packing. The two copies of RBD (Chains A/B, red) and DL28 (Chains C/D, blue) in an asymmetric unit interacts with DL28 (Chain D', green) and RBD (Chain B', cyan) molecules from adjacent units. (**B, C**) Expanded view of the crystal contacts near the DL28-RBD interaction surface. The crystal contacts for the two DL28-RBD copies are different. For Chain A/C (**B**), a DL28' molecule (DL28 from an adjacent asymmetric unit) binds the 'backrest' region of RBD and part of the DL28 framework region. For Chain B/D (**C**), a RBD' molecule (from an adjacent asymmetric unit) approaches from the opposite side of DL28. (**D**) Superposition of Chain A/C (red/blue) with Chain B/D (grey) shows nearly identical conformation between the two non-crystallography symmetry (NCS)-related copies of the DL28-RBD complex.

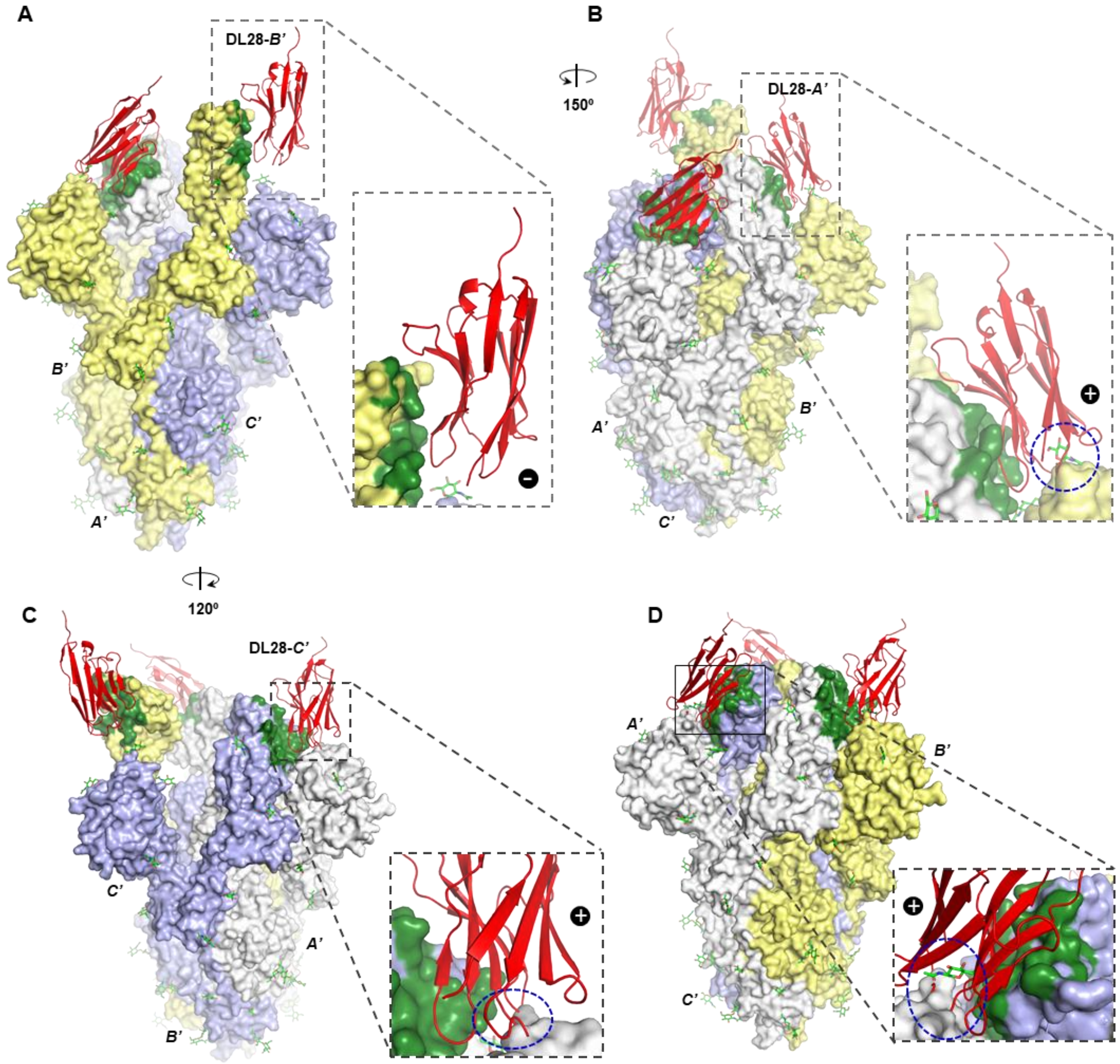

Supplementary Figure 2. The DL28 epitope is accessible in the 'up'-RBD. (**A-C**) DL28 is aligned to the open conformation of Spike (S) (PDB ID 6VYB) [1] on its 'up'-RBD (**A**) and two 'down'-RBDs (**B** and **C**). Three subunits are labeled with *A'*, *B',* and *C'*. DL28 docked to the subunits are marked with the individual *A'/B'/C'* subunits. '+' denote clashes (blue circle) and '-' denotes lack of clashes. (**D**) DL28 clashes with the closed conformation of S (PDB ID 6VXX) [1]. Because the three subunits in the closed conformation are identical, the interactions are only shown for one subunit.

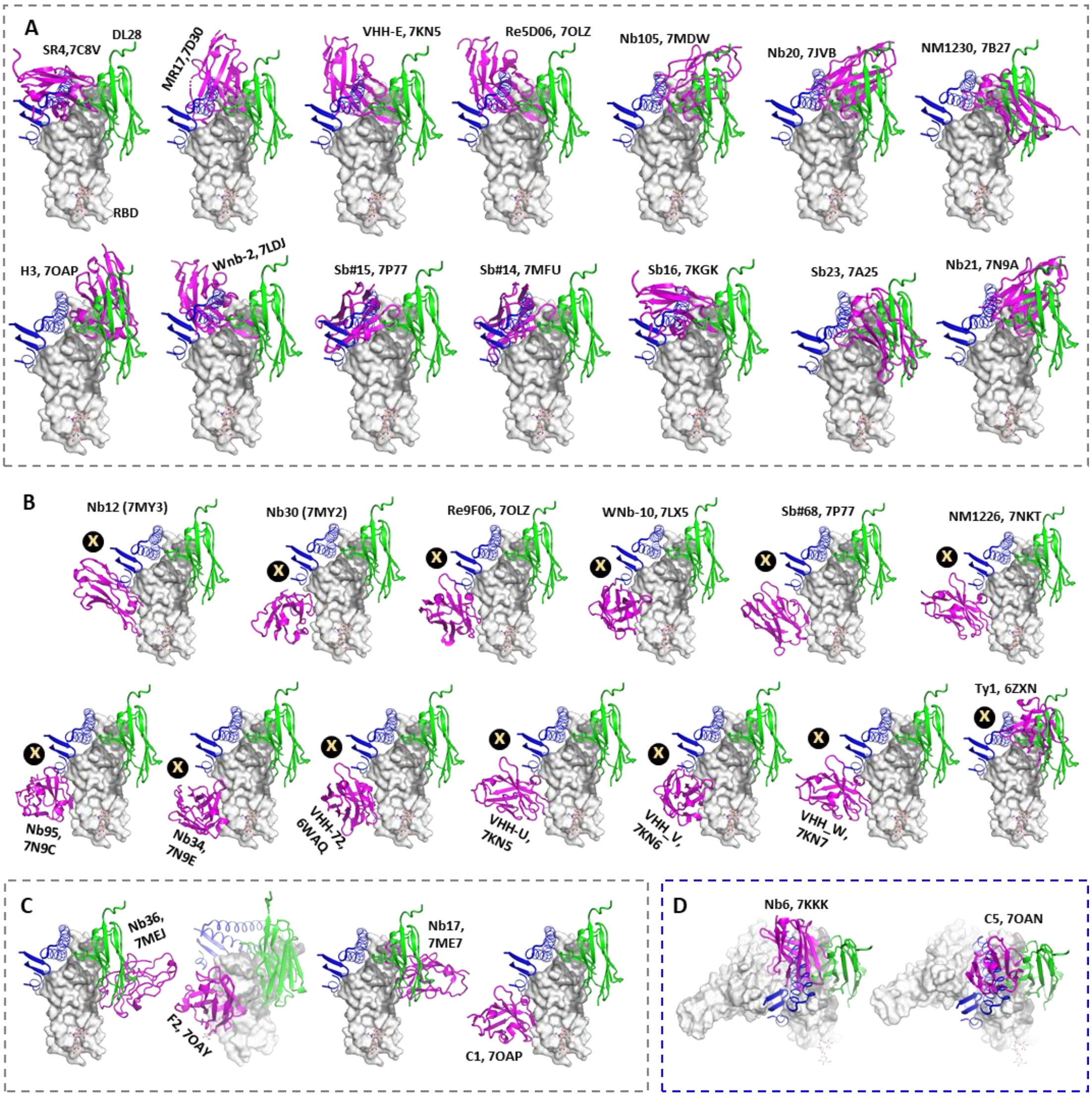

Supplementary Figure 3. Epitope comparison between DL28 and structurally characterized neutralizing nanobodies. (**A**) Comparison between DL28 and nanobodies that block ACE2 by direct competition. (**B**) Comparison between DL28 and nanobodies that block ACE2 by steric hindrance. (**C**) Comparison between DL28 and nanobodies that neutralize SARS-CoV-2 by destabilizing S. (**D**) Comparison between DL28 and nanobodies that lock RBD in the 'down' conformation. RBD is shown as a white surface. DL28 is shown as green and other nanobodies are shown as magenta cartoon. Part of the ACE2 that directly contacts RBD is shown as blue cartoon representation. In B, a cross-mark approximates steric hindrance. The nanobodies are labeled by their names followed by their PDB accession codes.

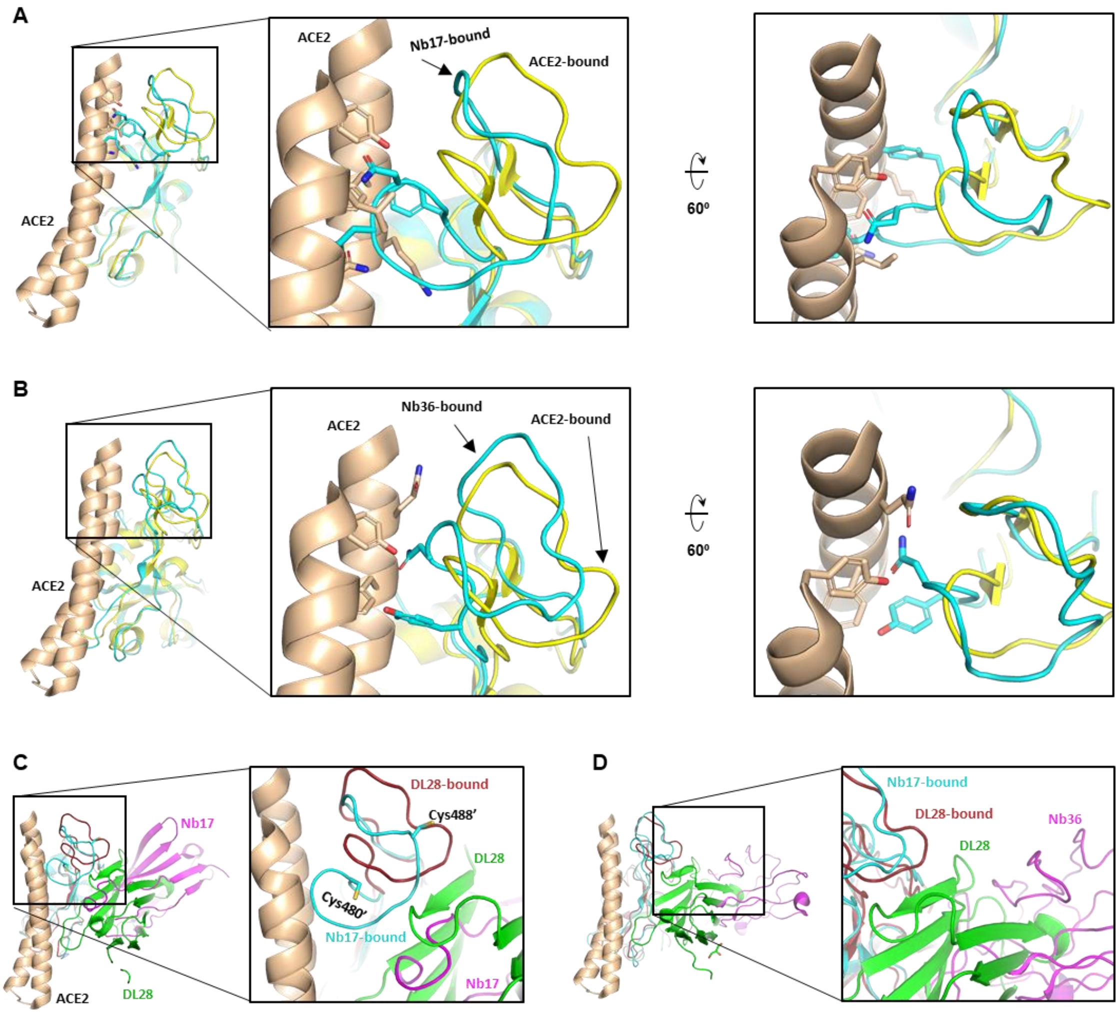

Supplementary Figure 4. Comparison between DL28 and two overlapping nanobodies (NB17 and NB36). (**A, B**) The 'backrest' region (**Figure 4A**) in the Nb17-bound form (cyan) (**A**) or in the Nb36-bound form (cyan) (**B**) differs from that in the ACE2-bound form (yellow) and clashes with ACE2 (wheat) in the aligned model. (**C**) Comparison between DL28-RBD (green and red) and Nb17-RBD (magenta and cyan). Two RBD-contacting helices of ACE2 are shown in wheat. The Cys480'-Cys488' disulfide pair is broken in Nb17-bound RBD. As a result, the loop deforms and is expected to be more flexible. (**D**) Comparison between DL28-RBD (green and red) and Nb36-RBD (magenta and cyan). Two RBD-contacting helices of ACE2 are shown in wheat. Unlike DL28, Nb36 does not interact directly with the 'backrest' region. In addition, Nb36 is remote from this region. Therefore, this region is expected to be able to move towards Nb36 and thus to avoid clashes with ACE2.

REFERENCES

1. Walls, A.C. et al. Structure, Function, and Antigenicity of the SARS-CoV-2 Spike Glycoprotein. *Cell* **181**, 281-292.e6 (2020).
